# Evaluation of antiarthritic activity of *Strychnos potatorum *Linn seeds in Freund's adjuvant induced arthritic rat model

**DOI:** 10.1186/1472-6882-10-56

**Published:** 2010-10-13

**Authors:** Sanmugapriya Ekambaram, Senthamil Selvan Perumal, Venkataraman Subramanian

**Affiliations:** 1 Department of Pharmaceutical Technology, Anna University of Technology, Tiruchirappalli -620 024, Tamilnadu, India; 2 C. L. Baid Mehta Foundation for Pharmaceutical Education & Research, Jyoti nagar, Old Mahabalipuram Road, Thorapakkam, Chennai - 600 096, Tamilnadu, India

## Abstract

**Background:**

*Strychnos potatorum *Linn (Loganiaceae) is a moderate sized tree found in southern and central parts of India, Sri Lanka and Burma. In traditional system of medicine, *Strychnos potatorum *Linn seeds were used for various ailments including inflammation, diabetes etc. To investigate the folkloric use of the seeds the present study was carried out on Freund's adjuvant induced arthritic rats.

**Methods:**

The present study states the effect of the aqueous extract (SPE) and the whole seed powder (SPP) of *Strychnos potatorum *Linn seeds on the Freund's complete adjuvant (FCA) induced arthritic rat paw edema, body weight changes and alterations in haematological and biochemical parameters in both developing and developed phases of arthritis. Histopathology of proximal interphalangeal joints and radiology of hind legs were studied.

**Results:**

In FCA induced arthritic rats, there was significant increase in rat paw volume and decrease in body weight increment, whereas SPP and SPE treated groups, showed significant reduction in paw volume and normal gain in body weight. The altered haematological parameters (Hb, RBC, WBC and ESR) and biochemical parameters (blood urea, serum creatinine, total proteins and acute phase proteins) in the arthritic rats were significantly brought back to near normal by the SPP and SPE treatment at the dose of 200 mg/kg/p.o in both developing and developed phases of arthritis. Further the histopathological and radiological studies revealed the antiarthritic activity of SPP and SPE by indicating fewer abnormalities in these groups when compared to the arthritic control group.

**Conclusion:**

In conclusion, both SPP and SPE at the specified dose level of 200 mg/kg, p.o. showed reduction in rat paw edema volume and it could significantly normalize the haematological and biochemical abnormalities in adjuvant induced arthritic rats in both developing and developed phases of FCA induced arthritis. Further the histopathological and radiological studies confirmed the antiarthritic activity of SPP and SPE.

## Background

Rheumatoid arthritis (RA) is a systemic autoimmune disease of unknown aetiology. The disease is characterized by articular inflammation and by the formation of an inflammatory and invasive tissue, rheumatoid pannus that eventually leads to the destruction of joints. Analgesia (painkillers) and anti-inflammatory drugs, including steroids are used to suppress the symptoms, while disease-modifying antirheumatic drugs (DMARDs), newer therapies such as anti-tumour necrosis factor (TNF)-α therapy (etanercept, infliximab and adalimumab), anti-CD20 therapy (rituximab) and abatacept are often required to inhibit or halt the underlying immune process. However, all of these agents are associated with numerous side effects. In recent days, researchers are directed towards traditional system of medicine for the discovery of drugs that are long acting anti-inflammatory with minimum side effects. Although there is no ideal animal model for RA at this time, rat adjuvant arthritis shares many features of human RA [1], and the sensitivity of this model to antiarthritic agents [2] support the view the adjuvant arthritis is the best available model of rheumatoid arthritis.

*Strychnos potatorum *Linn (Fam: Loganiaceae) is a moderate sized tree found in southern and central parts of India, Sri Lanka and Burma [3]. The root cures all kind of leucoderma. The ripe fruit is emetic, diaphoretic, alexiteric, cures inflammation, anemia, jaundice [4]. The seeds are used in hepatopathy, nephropathy, gonorrhoea, leucorrhoea, gastropathy, bronchitis, chronic diarrhoea, dysentery, renal and vesicle calculi, diabetes, burning sensation, dipsia, conjunctivitis, scleritis, ulcers and other eye diseases [5]. Phytochemical studies revealed the presence of diaboline (major alkaloid) and its acetate [6], triterpenes and sterols [7], mannogalactans [8]. The seeds are reported to have various activities like antidiabetic [9], antihypercholesterolemic activity [10], diuretic [11], antidiarrhoeal [12], hepatoprotective [13] and antiulcer [14].

Although the seeds possess many potential therapeutic activities in traditional system of medicinal practice and possessing rich phytoconstituents, they are not evaluated for their pharmacological activities in detail. The other species of the same genus, *Strychnos nuxvomica *was used in the treatment of rheumatoid arthritis and it was proved to be effective [15]. Taking these facts into considerations, the present study deals with the evaluation of antiarthritic activity and its changes in haematological and biochemical parameters of the aqueous extract and seed powder of *Strychnos potatorum *Linn seeds in Freund's adjuvant induced arthritic rats.

## Methods

### Collection of specimen (Strychnos potatorum Linn seeds)

The seed specimens for the proposed study were collected from crude drug market, Chennai and the genuinity of the seed specimen was confirmed by Dr. S. Jayaraman, Botanist, Plant Anatomy Research Centre, Chennai, Tamilnadu. A voucher specimen has been retained by the Department of Pharmacology and Environmental Toxicology (PET-02/2003), Dr.ALM PGIBMS, University of Madras, Chennai.

### Preparation of the extract

The air-dried seeds were coarsely powdered and subjected to hot water decoction for 2 hrs, it was then filtered and the filtrate was evaporated to dryness. A grey colored semisolid mass was obtained which was dried under vacuum and kept in a desiccator. The percentage yield of the extract (SPE) was 22.5% w/w from the starting crude material. The seed powder (SPP) as such was also used for the treatment. For the experimental study, both the drugs (SPP and SPE) were triturated with distilled water and administered immediately.

### Animals used

Wistar albino rats (140 ± 20 g) procured from TANUVAS (Tamilnadu University of Veterinary and Animal Sciences) was used for the study. The animals were kept in polypropylene cages and maintained at a temperature of 22 ± 2°C. They were fed with standard pellet feed (TANUVAS) and water *ad libitum*. The study has got the approval from the Institutional Animal Ethical Committee (IAEC) of CPCSEA (Committee for the Purpose of Control and Supervision of Experiments on Animals).

### Induction of arthritis

Arthritis was induced in rats by the intraplantar injection of 0.1 ml of Complete Freund's Adjuvant (CFA) in the left hind paw [16]. The adjuvant contained heat killed *Mycobacterium tuberculosis *(H_37_R_v _strain, Tuberculosis Research Centre, ICMR, Chennai) in sterile paraffin oil (10 mg/ml). The paw volume of all the animal groups was measured by plethysmograph at 0, 7, 14, 21 and 28 days after the injection of Freund's complete adjuvant.

### Experimental set up

Wistar albino rats of either sex weighing 140 ± 20 g were used for the study. The animals were divided into nine groups of six animals each. The standard drug Diclofenac sodium is dissolved in water whereas SPP and SPE were triturated with water in a glass mortar, made as a suspension and administered immediately. Group I served as control (without treatment), Group II served as arthritic control (negative control), Group III is treated with Diclofenac sodium (positive control) the standard anti - arthritic drug, Group IV & VII were treated with SPP and SPE for 28 days without inducing arthritis, Group V and VIII received SPP and SPE treatment for 28 days from the induction day (0-28 days treatment). Groups VI and IX received SPP and SPE for 14 days from the 14^th ^day of induction (14^th ^to 28^th ^day treatment). The body weight changes were observed on every week.

On 29^th ^day, at the end of experiment, all animals were sacrificed by cervical decapitation and blood was collected in plain and EDTA containing tubes, respectively for plasma/serum separation. The plasma/serum and homogenized samples were subjected to biochemical examination like total protein and albumin, globulin [17], acute phase proteins- Fibrinogen [18] Ceruloplasmin [19].

For histopathology, the proximal interphalangeal joints were removed, washed with saline and stored in 10% formalin. The interphalangeal joint sections were obtained, stained with eosin-haematoxylin stain and viewed under 100 X magnifications. The hind (arthritis induced) legs of the experimental rats were taken X-ray, and examined for the soft tissue swelling, bony erosions and narrowing of the spaces between joints.

### Statistical analysis

The data represents Mean ± S.E.M. Results were analyzed statistically by One-Way ANOVA followed by Tukey's multiple comparison using SPSS software student's version. The difference was considered significant when p < 0.05.

## Results

### FCA induced rat paw edema

There is a significant increase in rat paw volume in FCA injected arthritic control rats when compared to the normal control rats. SPP and SPE treatment at the dose of 200 mg/kg showed significant reduction in rat paw edema volume when compared with the arthritic group (table 1)

**Table 1:** Effect of SPP and SPE on rat paw edema in Freund's adjuvant induced arthritic rats

	Rat paw volume in ml ± S.E.M (% inhibition)				
	
Groups	Day 0	Day 7	Day 14	Day 21	Day 28
Group I	0.22 ± 0.05	0.25 ± 0.09	0.21 ± 0.05	0.22 ± 0.09	0.21 ± 0.08

Group II	0.27 ± 0.03	0.82 ± 0.05 a*	0.80 ± 0.09 a*	0.71 ± 0.12 a*	0.77 ± 0.09 a*

Group III	0.26 ± 0.01 (0.037%)	0.53 ± 0.03 a*b* (35.36%)	0.40 ± 0.05a* b* (50%)	0.42 ± 0.05 a*b* (40.84%)	0.38 ± 0.07 a^#^b* (50.64%)

Group IV	0.20 ± 0.07	0.23 ± 0.05 b*	0.25 ± 0.01 b*	0.26 ± 0.11 b*	0.22 ± 0.03b*

Group V	0.26 ± 0.06 (0.037%)	0.58 ± 0.04 a*b* (29.26%)	0.48 ± 0.08 a*b* (40%)	0.46 ± 0.09 a*b* (35.21%)	0.42 ± 0.02 a*b* (45.45%)

Group VI	0.24 ± 0.08 (11.11%)	0.62 ± 0.09 a*b* (24.39%)	0.52 ± 0.09 a*b* (35%)	0.52 ± 0.07 a*b* (26.76%)	0.50 ± 0.04 a*b* (35.06%)

Group VII	0.25 ± 0.04	0.25 ± 0.08 b*	0.26 ± 0.07 b*	0.26 ± 0.05 b*	0.21 ± 0.01b*

Group VIII	0.28 ± 0.09 (0.037%)	0.54 ± 0.05 a*b^# ^c^@^ (34.14%)	0.50 ± 0.09 a*b* c^#^ (37.5%)	0.49 ± 0.04 a*b*c^@^ (30.98%)	0.45 ± 0.11 a*b* (41.56%)

Group IX	0.24 ± 0.05 (11.11%)	0.67 ± 0.11 a*b* (18.29%)	0.54 ± 0.08 a* b* c^@^ (32.5%)	0.52 ± 0.08 a*b* (26.76%)	0.52 ± 0.10 a*b* (32.46%)

Values are Mean ± S.E.M, n = 6 animals in each group. Values in parenthesis indicate % inhibition of rat paw edema of treated groups Vs arthritic control group II.

Comparisons were made between: a- Group I vs II, III, IV, V, VI, VII, VIII and IX

b- Group II vs III, IV, V, VI, VII, VIII and IX

c- Group III vs V, VI, VIII and IX

Symbols represent statistical significance: * - p < 0.001, # - p < 0.01, @ - p < 0.05

### Body weight changes

**Figure 1:**
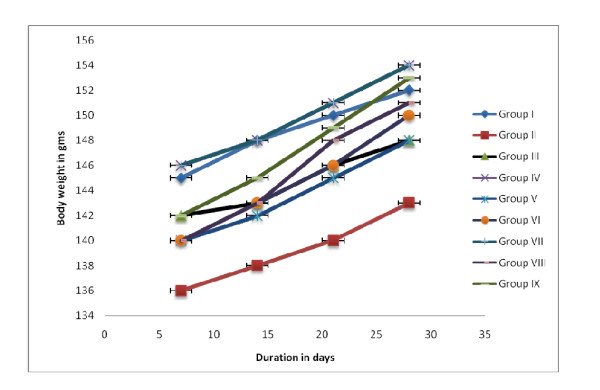
Relationship between the extent of joint inflammation and the degree of weight loss.

In the present study, it is clear from the data obtained (Fig. 1), that there is a close relationship between the extent of joint inflammation and the degree of weight loss. In the first week after adjuvant injection, the arthritic rats showed marked weight loss, followed by normal weight gain in the subsequent weeks, whereas the SPP, SPE and standard drug treated groups did not show any weight loss.

### Haematological parameters

The changes in hematological parameters in adjuvant induced arthritic rats are shown in table 2. There was a significant i) decrease in RBC count and haemoglobin, ii) increase in WBC count and ESR of arthritic rats, when compared with the control rats. The drug treatment has significantly brought back the altered haematological changes in both developing and developed phases of adjuvant induced arthritis.

**Table 2:** Effects of SPP and SPE on haematological parameters of control and adjuvant induced arthritic rats

Groups	RBC (millions/mm^3^)	WBC (thousands/mm^3^)	Hb (g/dl)	ESR (mm/hr)
Group I	5.84 ± 0.27	7.84 ± 0.34	14.22 ± 0.64	3.98 ± 0.07

Group II	4.95 ± 0.17 a*	12.94 ± 0.25 a*	11.54 ± 0.48 a*	10.56 ± 0.10 a*

Group III	5.78 ± 0.45 b*	8.75 ± 0.45 a^@^b*	14.34 ± 0.58 b*	5.32 ± 0.08 a^#^b*

Group IV	5.90 ± 0.18 b*	7.89 ± 0.34 b*	14.36 ± 0.64 b*	3.69 ± 0.14 b*

Group V	5.80 ± 0.14 b*	9.38 ± 0.48 a*b*	14.54 ± 0.27 b*	6.11 ± 0.16 a*b*

Group VI	5.79 ± 0.16 b*	10.65 ± 0.42 a*b*c^#^	13.98 ± 0.34 b*	6.85 ± 0.08 a*b*

Group VII	6.01 ± 0.27 b*	7.57 ± 0.39 b*	14.33 ± 0.58 b*	3.09 ± 0.10 b*

Group VIII	5.87 ± 0.29 b*	8.96 ± 0.15 a^@^b*	14.44 ± 0.34 b*	5.12 ± 0.14 a^#^b*

Group IX	5.81 ± 0.21 b*	9.14 ± 0.81 a*b*	14.65 ± 0.45 b*	5.70 ± 0.09 a^#^b*

Values are Mean ± S.E.M, n = 6 animals in each group.

Comparisons were made between: a- Group I vs II, III, IV, V, VI, VII, VIII and IX

b- Group II vs III, IV, V, VI, VII, VIII and IX

c- Group III vs V, VI, VIII and IX

Symbols represent statistical significance: * - p < 0.001, # - p < 0.01, @ - p < 0.05

### Blood glucose, urea and serum creatinine

Table 3 shows significant (p < 0.001) increase in the values of blood urea and serum creatinine in adjuvant induced arthritic rats when compared with the normal control group. These alterations were significantly reduced by treatment with SPP, SPE and Diclofenac sodium.

**Table 3:** Effects of SPP and SPE on Blood glucose, urea, creatinine and serum proteins of control and adjuvant induced arthritic rats

GROUPS	Urea (mg/dl)	Creatinine (mg/dl)	Protein (g/dl)	Albumin (g/dl)	Globulin (g/dl)	A/G ratio	Cerruloplasmin (mg/dl)	Fibrinogen (mg/dl)
Group I	22.64 ± 0.84	1.38 ± 0.06	6.62 ± 0.20	3.58 ± 0.06	2.30 ± 0.08	1.60 ± 0.10	19.65 ± 2.65	42.65 ± 2.28

Group II	30.54 ± 0.39 a*	2.38 ± 0.04 a*	4.28 ± 0.25 a*	2.36 ± 0.09 a*	2.86 ± 0.06 a*	0.79 ± 0.10 a*	52.74 ± 2.84 a*	162.45 ± 4.93 a*

Group III	23.15 ± 0.75 b*	1.67 ± 0.04 a^#^b*	5.32 ± 0.18 a*b*	3.02 ± 0.10 a*b*	2.54 ± 0.04 a*b*	1.15 ± 0.08 a*b*	22.12 ± 1.34 b*	88.04 ± 3.22 a*b*

Group IV	21.64 ± 0.83 b*	1.42 ± 0.03 b*	6.59 ± 0.19 b*	3.54 ± 0.12 b*	2.22 ± 0.06 b*	1.60 ± 0.11 b*	18.54 ± 1.24 b*	43.68 ± 4.14 b*

Group V	24.45 ± 0.64 b^#^	1.72 ± 0.08 a^#^b*	5.30 ± 0.20 a*b*	2.98 ± 0.08 a*b*	2.58 ± 0.08 a*b*	1.18 ± 0.07 a*b*	26.81 ± 2.46 a^@^b*	92.66 ± 5.64 a*b*

Group VI	25.88 ± 0.84 a^@^b^#^	1.79 ± 0.06 a*b*	5.24 ± 0.15 a*b*	2.89 ± 0.09 a*b*	2.62 ± 0.09 a*b*	1.12 ± 0.09 a*b*	26.24 ± 3.65 a^@^b*	98.53 ± 6.82 a*b*

Group VII	21.16 ± 1.28 b*	1.33 ± 1.06 b*	6.52 ± 0.19 b*	3.64 ± 0.07 b*	2.24 ± 0.04 b*	1.61 ± 0.04 b*	18.63 ± 2.94 b*	42.20 ± 7.87 b*

Group VIII	23.27 ± 0.84 b*	1.65 ± 0.06 a^#^b*	5.35 ± 0.16 a*b*	3.08 ± 0.06 a*b*	2.55 ± 0.06 a*b*	1.20 ± 0.08 a*b*	23.48 ± 2.45 b*	85.42 ± 8.60 a*b*

Group IX	24.84 ± 1.15 b^#^	1.68 ± 0.08 a^#^b*	5.30 ± 0.15 a*b*	2.97 ± 0.15 a*b*	2.58 ± 0.07 a*b*	1.18 ± 0.05 a*b*	24.35 ± 3.40 a^@^b*	93.60 ± 5.40 a*b*

Values are Mean ± S.E.M, n = 6 animals in each group.

Comparisons were made between: a- Group I vs II, III, IV, V, VI, VII, VIII, IX

b- Group II vs III, IV, V, VI, VII, VIII, IX

c- Group III vs V, VI, VIII, IX

Symbols represent statistical significance: * - p < 0.001, # - p < 0.01, @ - p < 0.05

in adjuvant induced arthritic rats.

### Total protein, albumin, globulin and A/G ratio

The changes in total and individual protein levels are presented in table 3. In adjuvant injected arthritic rats, there was significant decrease (p < 0.001) in total protein, albumin levels and A/G ratio, but significant (p < 0.001) increase in globulin level on comparison with the control group. On treatment with SPP, SPE and Diclofenac sodium all these changes were brought back to normal.

### Acute phase proteins

Fibrinogen and ceruloplasmin are regarded as acute phase proteins. In arthritis-induced rats, these two acute phase markers were significantly (p < 0.001) increased when compared with the arthritic control group (Table 3). Treatment with SPP and SPE have significantly (p < 0.001) decreased the levels of acute phase proteins in arthritic rats.

## Discussion

Paw swelling is an index of measuring the antiarthritic activity of various drugs and it is employed here to determine the activity of SPP and SPE at the dose level 200 mg/kg/p.o. SPP and SPE administered groups showed marked reduction in paw volume when compared with the arthritic control group. Yoshikawa *et al*., [20] found that there was significant weight loss, the day following the injection of the adjuvant, but thereafter continued to show normal weight gain in rats. The result of the present study also indicates that there is a close relationship between the extent of inflammation and loss of body weight.

From the results it is clear that the decrease in RBC count and haemoglobin level represents the anemic condition in arthritic rats. The more important causes are the abnormal storage of iron in the reticuloendothelial system and synovial tissue and the failure of bone marrow to respond to anemia [21]. The significant increase in leukocyte count in adjuvant-induced arthritic rats may be due to the stimulation of immune system against the invading antigens and the respective decrease in SPP and SPE treated groups showed its immunomodulation effect. The ESR count which significantly increased in arthritic control group has been remarkably counteracted by SPP, SPE and standard drug Diclofenac sodium, restoring back to near normal thus justifying its significant role in arthritic conditions.

Increased blood urea and serum creatinine level also found in the arthritic control group which indicates the kidney dysfunction in arthritic rats. SPP and SPE treatment has significantly reduced the altered urea and serum creatinine levels. Increased blood urea level was reported in arthritic rats and it was hypothesized that substantial fraction of blood urea in arthritic rats comes from arginine synthesized in the kidneys [22].

In the present study there was a significant decrease in albumin level and increase in globulin level in arthritic rats. The adjuvant induced arthritis causes changes in plasma protein concentrations that are manifested as an increase in the globulin fraction and decrease in the albumin fraction [23]. It was also postulated that during inflammation, the mediators released, histamine, bradykinin and prostaglandins increase the permeability of vascular tissues to albumin leading to reduction in its serum levels [24]. Thus treatment with SPP and SPE could significantly increase the albumin and decreased the globulin level in arthritic rats which indicates that SPP and SPE might have a suppressive action on the mediators of inflammation.

The measurement of acute phase proteins in plasma provides a clinically valuable indication of the presence of inflammation and its extent [25]. Ceruloplasmin, a copper containing plasma protein is produced in liver in response to tissue injury and released into circulation [26]. Thus, its elevated serum level during chronic arthritis might indicate continued tissue injury [27]. In the present study, the increased acute phase proteins fibrinogen and ceruloplasmin were significantly decreased by the SPP and SPE treatment, which shows its effect in tissue repair.

**Figure 2:**
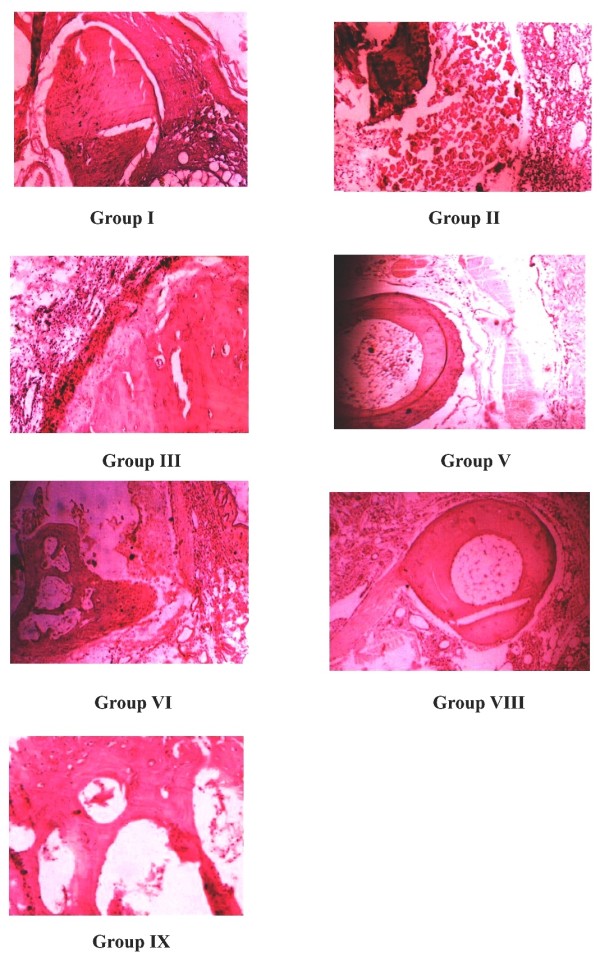
**Histopathology of proximal interphalangeal joints on adjuvant induced arthritic rats**.

The observed histopathological changes of proximal interphalangeal joints of the experimental groups are shown in fig. 2. Group I showed the histopathology of normal ankle joint. Group II the negative control arthritic rat joint showed prominent abnormalities from the normal joint like edema formation, degeneration with partial erosion of the cartilage, destruction of bone marrow and extensive infiltration of inflammatory exudates in the articular surface. The standard drug treated rat joint showed normal bone marrow with less cellular infiltrates. SPP and SPE treatment for 28 days showed less inflammatory signs like scanty cellular infiltrate, absence of edema formation and normal bone marrow, whereas the SPP and SPE treatment for 14 days showed cellular infiltrates on the articular surface with less cartilage destruction. The overall prevention of the inflammatory signs of the rat ankle joints was significant in 28 days drug treated group compared with 14 days drug treated group. Degeneration of the ankle joint was not observed in any of the drug treated groups when compared with the negative control.

**Figure 3:**
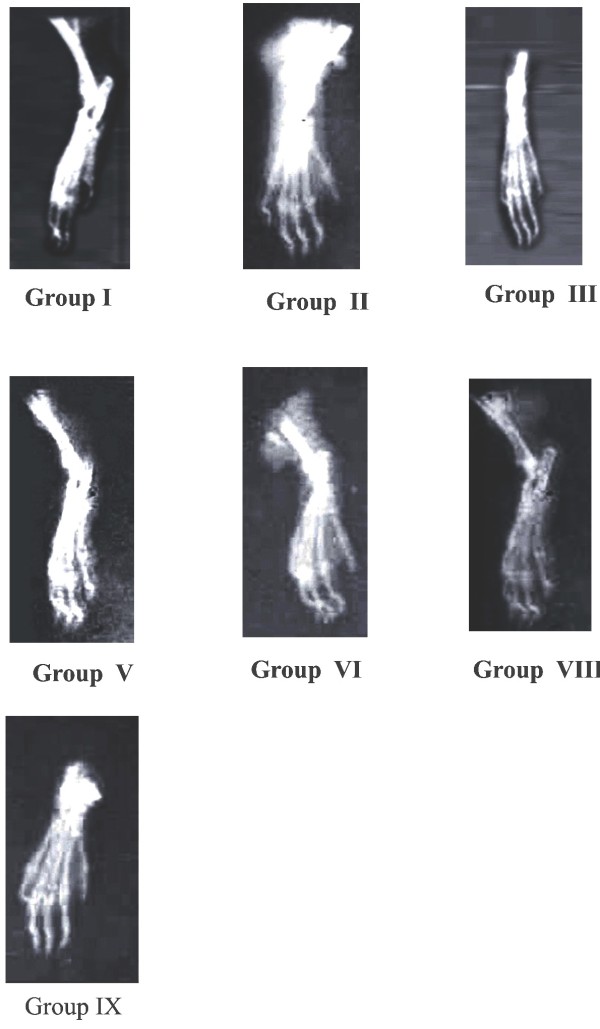
**Radiology of hind legs in adjuvant induced arthritic rats**.

Radiographic changes in RA conditions are useful diagnostic measures which indicate the severity of the disease. Soft tissue swelling is the earlier radiographic sign, whereas prominent radiographic changes like bony erosions and narrowing of joint spaces can be observed only in the developed stages (final stages) of arthritis [28]. The radiographic features of the rat joints in adjuvant induced arthritic model are shown in fig. 2. In adjuvant induced arthritic rat (group II), soft tissue swelling along with narrowing of the joint spaces were observed which implies the bony destruction in arthritic condition. The standard drug Diclofenac sodium treated groups have prevented this bony destruction and also there is no swelling of the joint. Similar to histopathological studies, SPP and SPE treatment for 28 days have shown significant prevention against bony destruction by showing less soft tissue swelling and narrowing of joint spaces when compared with 14 days SPP and SPE treated groups.

## Conclusion

In conclusion, both SPP and SPE at the specified dose level of 200 mg/kg, p.o. showed reduction in rat paw edema volume and it could normalize the haematological and biochemical abnormalities in adjuvant induced arthritic rats in both developing and developed phases of FCA induced arthritis. Further the histopathological and radiological studies confirmed the antiarthritic activity of SPP and SPE in FCA induced arthritis. The actual mechanism of action of SPP and SPE on adjuvant induced arthritis is not clear with these studies. The action of SPP and SPE on proinflammatory mediators like TNF- α, Interleukins and other relevant mediators will be carried out in future to study its mechanism.

## Competing interests

The authors declare that they have no competing interests.

## Authors' contributions

ES performed the whole study. PS contributed in conducting animal studies and analysis of biochemical parameters. SV participated in the design of the study. All authors read and approved the final manuscript.

## Pre-publication history

The pre-publication history for this paper can be accessed here:

http://www.biomedcentral.com/1472-6882/10/56/prepub
